# Subclinical myocardial injury and cardiovascular mortality: Racial differences in prevalence and risk (from the third National Health and Nutrition Examination survey)

**DOI:** 10.1111/anec.12827

**Published:** 2021-03-06

**Authors:** Stephen T. Broughton, Muhammad Ahmad, Elsayed Z. Soliman, Jared W. Magnani

**Affiliations:** ^1^ Department of Internal Medicine Division of Cardiology University of Pittsburgh School of Medicine and Heart & Vascular Institute University of Pittsburgh Medical Center Pittsburgh Pennsylvania; ^2^ Department of Internal Medicine Wake Forest School of Medicine Winston‐Salem North Carolina; ^3^ Section on Cardiology Wake Forest School of Medicine Winston‐Salem North Carolina; ^4^ Department of Epidemiology and Prevention Epidemiological Cardiology Research Center (EPI‐CARE) Wake Forest School of Medicine Winston‐Salem North Carolina

**Keywords:** cardiovascular mortality, NHANES, racial differences, subclinical myocardial injury

## Abstract

**Background:**

Subclinical myocardial injury (SCMI) determined from the Electrocardiographic Cardiac Infarction/Injury Score (CIIS) is associated with increased risk of cardiovascular disease and mortality. We hypothesized that SCMI prevalence and association with mortality would differ by race, categorized as non‐Hispanic White (White), non‐Hispanic Black (Black), and Mexican American.

**Methods:**

Our analysis included 5,852 participants (age 58.5 ± 13.2 years; 54% women, 52% Whites, 23% Blacks, and 25% Mexican American participants) from the National Health and Nutrition Examination Survey (NHANES III, 1988–94) who were free of cardiovascular disease at the time of enrollment. SCMI was defined as the presence of CIIS ≥ 10 score points on the 12‐lead ECG. Prevalence of SCMI and its association with cardiovascular mortality were examined in each race/ethnic group in models adjusted for sociodemographics and common cardiovascular risk factors.

**Results:**

SCMI prevalence was 23.4% in Whites, 21.8% in Blacks, and 18.0% in Mexican Americans. Compared to Whites, Blacks were as likely to have SCMI (odds ratio [OR] 0.95, 95% confidence interval [CI] 0.80–1.13), while Mexican Americans were less likely (OR 0.74, 95% CI 0.62–0.88). SCMI was not associated with increased risk of cardiovascular mortality in either Whites (hazard ratio [HR] 1.18, 95% CI 0.95–1.48) or Blacks (HR 1.19, 95% CI 0.79–1.80). In contrast, SCMI in Mexican Americans was associated with increased risk of cardiovascular mortality (HR 1.74, 95% CI 1.13–2.67, *p* < .05).

**Conclusion:**

Mexican Americans had a lower prevalence of SCMI, but increased risk of cardiovascular mortality. Screening for SCMI may identify individuals at increased risk and improve targeted prevention efforts.

## 1 INTRODUCTION

The Cardiac Infarction Injury Score (CIIS) was developed in 1981 as an ECG classification to improve diagnostic accuracy of myocardial injury compared to conventional criteria (e.g., the Minnesota Code). CIIS uses a combination of features measured on a continuous scale to evaluate 12 items across multiple leads of a standard electrocardiogram (e.g., T‐wave amplitude and Q‐wave duration). (Rautaharju et al., 1981) Increased CIIS scores have been associated with identifying individuals at high risk for cardiovascular disease (CVD) mortality. (Dekker et al., 1994; Domburg et al., 1998; Richardson et al., 2005; Siscovick et al., 1996) While the prognostic ability of CIIS among a diverse US population without established CVD has been previously studied, there has been limited assessment of racial differences in the significance of subclinical myocardial injury (SCMI). (ONeal et al., 2014) Therefore, the purpose of this study was to evaluate SCMI prevalence and association with CVD mortality among different races using data from the Third National Health and Nutrition Examination Survey (NHANES III).

## 2 METHODS

NHANES is a periodic survey that samples a cohort representative of the US population and was designed to estimate disease prevalence and risk factors. The survey methods have been well described previously. (NHANES III, 1988–94) Briefly, baseline data for NHANES III were collected from an in‐home interview and a subsequent visit to a mobile examination center between 1988 and 1994. The interview portion includes demographic, medication information, socioeconomic, dietary, and health‐related questions, and the examination component consists of medical, dental, and physiologic measurements, as well as laboratory tests. All participants gave written informed consent at the time of study enrollment. Participant characteristics, electrocardiography methodology, and ascertainment of mortality in NHANES III have been previously published. (Yang et al., 2012) Blood samples were obtained at mobile centers, and basic laboratory values were recorded for each participant (total cholesterol, high‐density lipoprotein cholesterol, and plasma glucose). The present analysis included 5,852 participants free of baseline CVD and who had available baseline demographic, laboratory, medication, and mortality data. Participants with baseline CVD were excluded, determined by a self‐reported history of heart attack and/or stroke, electrocardiographic evidence of myocardial infarction, or major ST‐T depression by Minnesota Electrocardiogram Classification (Prineas et al., 2010).

Age, sex, race/ethnicity, smoking history, and household income were self‐reported. Medication history, including the use of antihypertensive, antidiabetic, and lipid‐lowering medications, was also self‐reported. Smoking was defined as ever or never smoker. Blood pressure measurements were obtained, and the average reading from 3 in‐home measurements and 3 mobile center measurements was used. Body mass index was computed as the weight in kilograms divided by the square of the height in meters. Obesity was defined as a body mass index ≥ 30 kg/m^2^. Diabetes was defined as a fasting plasma glucose level ≥ 126 mg/dl, glycosylated hemoglobin A1c value ≥ 6.5, or a history of antidiabetic medication use. (NHANES III, 1988–94).

Standard 12‐lead electrocardiograms were recorded by trained technicians using a Marquette MAC 12 system (Marquette Medical Systems, Milwaukee, Wisconsin) during each participants’ visit to a mobile examination center. Computerized automated analysis of the electrocardiographic data was performed with visual inspection of outlier values by a trained technician in a central electrocardiographic core laboratory. The calculation of CIIS and methodology has been previously described. (Rautaharju et al., 1981) Briefly, the score is defined by a set of 10 discrete binary (e.g., a single threshold) and ternary (e.g., high and low threshold) features in combination with 2 features measured in continuum (linear numerical values), and provides a simple scoring scheme suitable for both visual and computer classification of the conventional 12‐lead electrocardiogram. Features utilized to calculate CIIS scores include Q‐wave duration and amplitude, R‐wave amplitude, and T‐wave amplitude, among others, in various precordial and limb leads. The CIIS values were multiplied by a factor of 10 in NHANES III to avoid using decimal points. We divided the reported CIIS values by 10 to remain consistent with prior studies. (ONeal et al., 2014) Subclinical myocardial injury was defined as CIIS values ≥ 10, representing the limit for abnormal CIIS. (Rautaharju et al., 1981).

Mortality data for NHANES III participants were available through December 31, 2006, and methods for mortality ascertainment have been described. (Statistics NCFH, 2006) A probabilistic matching algorithm based on 12 identifiers was used to link participants with death information captured in the National Death Index. Matching identifiers included social security number, sex, and date of birth. Follow‐up was defined as the interval between the NHANES III examination and either of the following, depending on which came first: date of death, date of censoring, or December 31, 2006. The end point of CVD mortality was examined and analyzed using data from the NHANES III Linked Mortality File. *International Classification of Diseases, Ninth Revision*, codes were used to identify the end point. CVD mortality was defined by codes 100 to 178. Participants who were unable to be matched with a death record were categorized as alive throughout follow‐up.

Continuous variables were reported as mean ± standard deviation and categorical variables by their distributions. Statistical significance for continuous variables was tested using the *t test* procedure and the Rao‐Scott chi‐square method for categorical variables. Unadjusted CVD mortality rates (per 1,000 person‐years) were calculated. Kaplan–Meier estimates were used to compute unadjusted survival estimates for CVD mortality, and the differences in estimates between presence and absence of SCMI were compared using the log‐rank procedure. (Gray & Tsiatis, 1989) Cox proportional hazards regression was used to generate hazard ratios (HR) and 95% confidence intervals (95% CI) for the association between SCMI and CVD mortality. Multivariable‐adjusted models were constructed with incremental adjustments as follows: model 1 unadjusted; model 2 adjusted for age, sex, and total annual income; and model 3 adjusted for model 2, plus smoking, hypertension, diabetes, hyperlipidemia, and obesity. Statistical significance was defined as *p* ≤ .05. Data were analyzed using SAS, version 9.3 (SAS Institute Inc).

## 3 RESULTS

A total of 5,852 participants (age 58.5 ± 13.2 years; 54% women, 52% non‐Hispanic Whites, 23% non‐Hispanic Blacks, and 25% Mexican Americans) were included in the analysis. The average CIIS values by race and sex were obtained (Whites 5.6 ± 7.0, Blacks 5.2 ± 6.7, Mexican Americans 4.5 ± 6.3; men 5.6 ± 7.0, women 4.9 ± 6.6). Baseline characteristics for study participants stratified by race/ethnicity, and then, SCMI are shown in Table 1. Participants with SCMI were more likely to be older, male, diabetic, obese, and take antihypertensive medications, compared with those without SCMI. Additionally, SCMI was associated with higher values of average baseline systolic blood pressure. White participants regardless of SCMI presence had a higher average baseline age and total cholesterol, and lower percentages of annual income <$20,000, compared to Black and Mexican American participants. Estimated prevalence of SCMI was 23.4% in Whites, 21.8% in Blacks, and 18.0% in Mexican Americans.

**TABLE 1:** Baseline Characteristics of the 5,852 Study Participants from NHANES III

Characteristics *Mean ± SD or n (%)*	Non‐Hispanic Whites			Non‐Hispanic Blacks			Mexican Americans		
	SCMI^a^ Absent *n* = 2,355	SCMI^a^ Present *n* = 719	*p*‐value*	SCMI^a^ Absent *n* = 1,045	SCMI^a^ Present *n* = 292	*p*‐value*	SCMI^a^ Absent *n* = 1,181	SCMI^a^ Present *n* = 260	*p*‐value*
Age (years)	60.3 ± 13.6	65.0 ± 13.1	<.001	54.2 ± 11.5	58.7 ± 12.0	<.001	54.7 ± 11.5	58.6 ± 11.9	<.0001
Men (%)	1,038 (44.0%)	342 (47.5%)	.009	439 (42.0%)	150 (51.3%)	.004	581 (49.2%)	145 (55.7%)	.05
Systolic Blood Pressure (mm Hg)	130.0 ± 18.6	134.5 ± 19.3	<.001	132.1 ± 19.6	135.0 ± 19.9	.02	129.8 ± 18.7	134.4 ± 17.8	.0003
Diastolic Blood Pressure (mm Hg)	75.5 ± 9.4	75.1 ± 9.6	.32	78.8 ± 10.8	77.0 ± 11.1	.12	75.8 ± 9.6	77.3 ± 10.7	.03
Antihypertensive (%)	452 (19.1%)	185 (25.7%)	.0002	254 (24.3%)	103 (35.2%)	.0002	147 (12.4%)	45 (17.3%)	.03
Diabetes (%)	160 (6.7%)	96 (13.3%)	<.001	139 (13.3%)	41 (14.0%)	.74	182 (15.4%)	67 (25.7%)	<.0001
Anti‐diabetics (%)	86 (3.6%)	50 (6.9%)	.0002	82 (7.8%)	29 (9.9%)	.25	112 (9.4%)	39 (15.0%)	.008
Total Cholesterol (mg/dl)	220.4 ± 42.0	222.1 ± 44.5	.37	213.3 ± 45.1	217.2 ± 48.5	.20	213.8 ± 41.8	219.4 ± 45.1	.05
Serum Triglycerides (mg/dl)	156.2 ± 138.6	167.8 ± 112.4	.02	127.6 ± 96.7	132.4 ± 98.5	.45	178.6 ± 124.0	201.2 ± 149.2	.02
Anti‐hyperlipidemic (%)	89 (3.7%)	22 (3.0%)	.36	19 (1.8%)	4 (1.3%)	.60	20 (1.6%)	10 (3.8%)	.02
Ever Smokers (%)	1,285 (54.5%)	449 (62.4%)	.002	580 (55.5%)	179 (61.3%)	.07	599 (50.7%)	145 (55.7%)	.14
Obesity (%)	515 (21.8%)	183 (25.4%)	.04	352 (33.6%)	101 (34.5%)	.77	379 (32.2%)	109 (42.0%)	.002
Annual Income below < 20 K (%)	706 (30.3%)	290 (40.8%)	<.0001	544 (53.1%)	173 (60.9%)	.01	699 (59.9%)	161 (63.6%)	.27

Note

Abbreviations: NHANES, National Health and Nutrition Examination Survey; SCMI, subclinical myocardial injury.

^a^ Subclinical myocardial injury defined by CIIS ≥ 10.

* p‐value calculated by *t* test or chi‐square.

**FIGURE 1:**
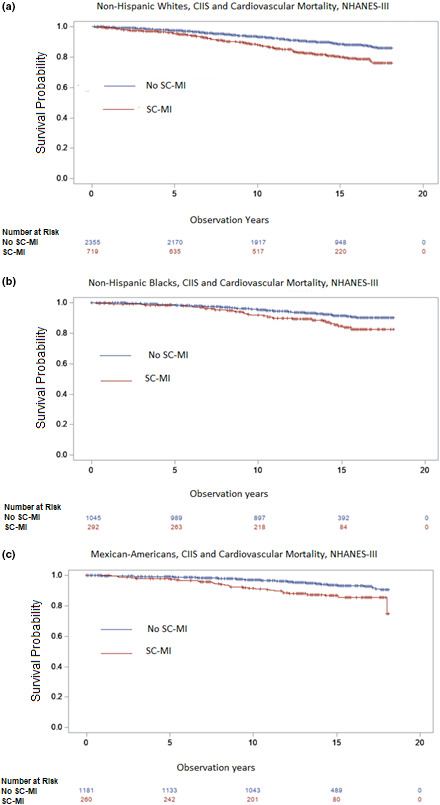
Unadjusted Kaplan–Meier survival estimates for mortality by subclinical myocardial injury among non‐Hispanic Whites (a), non‐Hispanic Blacks (b), and Mexican Americans (c). Mortalities were significantly different (log‐rank *p* < .005). Subclinical myocardial injury was defined by CIIS ≥ 10. CIIS, Cardiac Infarction/Injury Score; NHANES, National Health and Nutrition Examination Survey; SCMI, Subclinical Myocardial Injury. Color should be used for the print of Figure 1

Table 2 shows the risk of SCMI by race/ethnicity in unadjusted and multivariable‐adjusted regression models. Compared to Whites, the likelihood of SCMI among Black (OR 0.95, 95% CI 0.80–1.13, *p* = .62) did not differ significantly. Mexican Americans had decreased likelihood of SCMI (OR 0.74, 95% CI 0.62–0.88, *p* < .05) compared to Whites, which persisted after multivariable adjustment. Over a median follow‐up of 14 years, there were 581 CVD deaths. There were higher incident rates of CVD mortality in the presence of SCMI among all 3 race/ethnicity groups. Unadjusted Kaplan–Meier survival estimates are shown for CVD mortality among Whites, Blacks, and Mexican Americans in Figure 1.

**TABLE 2:** ORs and 95% CI association of race with SCMI^a^

Race	Model 1^b^ OR (95% CI)	*p*‐value	Model 2^c^ OR (95% CI)	*p*‐value	Model 3^d^ OR (95% CI)	*p*‐value
Non‐Hispanic Whites	Ref	‐	Ref	‐	Ref	‐
Non‐Hispanic Blacks	0.91 (0.78–1.06)	.26	1.03 (0.87–1.21)	.71	0.95 (0.80–1.13)	.62
Mexican American	0.72 (0.61–0.84)	<.0001	0.78 (0.65–0.92)	.004	0.74 (0.62–0.88)	.0009

Note

Abbreviations: CI, confidence interval; OR, odds ratio; SCMI, subclinical myocardial injury.

^a^ OR presented for subclinical myocardial injury defined by CIIS ≥ 10.

^b^ Model 1 unadjusted.

^c^ Model 2 adjusted for age, sex, and total annual income.

^d^ Model 3 adjusted for model 2 covariates plus smoking, hypertension, diabetes, hyperlipidemia, and obesity.

Table 3 lists hazard ratios demonstrating the association of SCMI with CVD mortality stratified by race. When compared to each respective race/ethnicity in the absence of SCMI, the presence of SCMI among non‐Hispanic Whites was associated with HR 1.18 (95% CI 0.95–1.48, *p* = .12), among non‐Hispanic Blacks was associated with HR 1.19 (95% CI 0.79–1.80, *p* = .38), and among Mexican Americans was associated with HR 1.74 (95% CI 1.13–2.67, *p* = .01). The increased risk of CVD mortality among Mexican Americans with SCMI remained statistically significant in the fully adjusted model.

**TABLE 3:** HRs and 95% CI association of SCMI^a^ with CVD mortality by race

Race	SCMI	Participants *n*	Events *n* (%)	Model 1^b^ HR (95% CI)	*p*‐value	Model 2^c^ HR (95% CI)	*p*‐value	Model 3^d^ HR (95% CI)	*p*‐value
Non‐Hispanic Whites	Absent	*2,355*	242 (10.2%)	Ref	‐	Ref	‐	Ref	*‐*
	Present	719	122 (16.9%)	1.85 (1.49–2.30)	<.0001	1.26 (1.01–1.57)	.03	1.18 (0.95–1.48)	.12
Non‐Hispanic Blacks	Absent	1,045	79 (7.5%)	Ref	‐	Ref	‐	Ref	*‐*
	Present	292	35 (11.9%)	1.79 (1.20–2.66)	.004	1.28 (0.85–1.92)	.23	1.19 (0.79–1.80)	.38
Mexican American	Absent	1,181	71 (6.0%)	Ref	‐	Ref	‐	Ref	*‐*
	Present	260	32 (12.3%)	2.27 (1.49–3.45)	.0001	1.77 (1.16–2.71)	.008	1.74 (1.13–2.67)	.01

Note

Abbreviations: CI, confidence interval; CVD, cardiovascular disease; HR, hazard ratio; SCMI, subclinical myocardial injury.

Interaction p‐value = 0.25.

^a^ HR presented for subclinical myocardial injury defined by CIIS ≥ 10.

^b^ Model 1 unadjusted.

^c^ Model 2 adjusted for age, sex, and total annual income.

^d^ Model 3 adjusted for model 2 covariates plus smoking, hypertension, diabetes, hyperlipidemia, and obesity.

## 4 DISCUSSION

In a large, diverse sample of US adults, the estimated prevalence of SCMI determined by CIIS values (CIIS ≥ 10) was highest among non‐Hispanic Whites, followed by non‐Hispanic Blacks, and then Mexican Americans. When compared to Whites, Blacks were just as likely to have SCMI, while Mexican Americans were less likely. After adjusting for potential confounding factors, the presence of SCMI in adults without baseline CVD was associated with an increased risk of CVD mortality in Mexican Americans, but not Whites or Blacks.

Prior studies have evaluated the prognostic significance of SCMI as determined by CIIS. (Dekker et al., 1994; Domburg et al., 1998; ONeal et al., 2014; Richardson et al., 2005; Siscovick et al., 1996) In a large cohort of apparently healthy men and women in the Netherlands, elevated CIIS values were associated with significantly higher rates of coronary heart disease and CVD mortality. (Dekker et al., 1994) In a Washington state population‐based case–control study of patients free of clinical CVD, increasing CIIS values were linked with a higher risk of primary cardiac arrest among hypertensive patients. (Siscovick et al., 1996) High CIIS values (≥20) in a large series of postmyocardial infarction patients had an increased relative risk of total and CVD mortality at 1‐ and 3‐year follow‐ups. (Domburg et al., 1998) Additionally, CIIS was shown to be an independent risk factor for total and CVD mortality among a large population of US citizens without any self‐reported or electrocardiogram evidence of underlying CVD. (ONeal et al., 2014).

While there have been several studies that evaluated the ability of CIIS to prognosticate a risk of mortality in absence of established CVD, few have explored a large, racially and ethnically diverse population of US citizens. Our study shows that there are not only significant racial differences in the prognostic value of SCMI determined by CIIS, and there are also differences in its prevalence and association with each represented race as well. A prior study of a similar population showed an increased amount of CVD‐related deaths among non‐White individuals when compared to White individuals; however, the specific delineation of each non‐White race was not identified. (ONeal et al., 2014) Additionally, prevalence and association between SCMI and race has not been investigated among a similar cohort.

Surveillance data have suggested that there are racial and socioeconomic disparities in the prevalence, morbidity, and mortality associated with CVD and major risk factors. For example, non‐Hispanic Blacks have higher rates of CVD mortality and prevalence of hypertension, compared to non‐Hispanic Whites and Mexican Americans. Additionally, the highest prevalence of obesity among men was in Mexican Americans. (Mensah et al., 2005) Despite having a similar association with increasing rates of hypertension and obesity, our findings showed the highest prevalence of SCMI among those of a non‐Hispanic White background. While the total number of survey participants who identified as White more than doubled the number of Mexican Americans (*n* = 3,074 vs. *n* = 1,441), the association between SCMI and CVD mortality remained greater in the Mexican American group. Prior studies estimating the prevalence of CVD and prognostication of risk profiles among US Hispanics from NHANES data found similar results; lower overall prevalence of CVD compared to non‐Hispanic Whites and Blacks, but a higher association with adverse events. (Rodriguez et al., 2014) It is unclear exactly what additional factors may have influenced the results in our study, as there were no significant differences in baseline characteristics between race groups, and common confounders, such as demographics and traditional risk factors, were accounted for. Potential racial differences in access to care, proficiency of the English language, and health literacy were not explored among the study population, but these attributes are very likely to be playing a role in our results. It is also possible that there is a genetic component to the increased risk of CVD mortality in the presence of SCMI that may expose those of Mexican American background more than the other races in this study. Other previously established CVD risk factors, such as socioeconomic status (i.e., level of education, household income categorized by taxation rates, marital status, and others) were not included in this study. The inclusion of these additional socioeconomic factors might have also contributed to explaining our results, as there has been establishment of racial disparities within these categories. (Bell et al., 2018).

The electrocardiogram is a relatively inexpensive and noninvasive method of screening patients for underlying CVD that is regularly obtained. CIIS has been proven to accurately detect SCMI and has utility in prognostication of mortality among individuals without baseline CVD. (Dekker et al., 1994; Domburg et al., 1998; ONeal et al., 2014; Rautaharju et al., 1981; Richardson et al., 2005; Siscovick et al., 1996) From the results of our study, we have identified that Mexican Americans may be at a greater risk when SCMI is present, regardless of traditional risk factors. Thus, a more aggressive approach to CVD prevention and risk factor modification (i.e., consideration of pharmacological therapy, dietary and exercise recommendations, weight loss, and behavioral/lifestyle interventions) might prove protective in this vulnerable population. Similar results have been found in prior studies showing racial differences in adverse CVD events when utilizing a noninvasive method of assessing CVD risk. For example, Li et al found that minor isolated Q waves in Hispanics without baseline CVD was significantly associated with incident CVD events, but not in non‐Hispanic Whites or Blacks. (Li et al., 2013) SCMI represents a separate risk factor with similar racial differences that may have been previously overlooked, but yet could be clinically valuable. Race‐specific cut points have not been established for CIIS and are beyond the scope of this analysis. It is premature to consider race‐specific values for CIIS simply based on these findings. Further areas of research include assessment of the optimal CIIS cutoff value to define SCMI in each race, and if the ECG variables included in the scoring should differ by race.

The strengths of this study include population‐based sampling, a large number of minority participants frequently underrepresented in clinical trials and registries, validated assessments, and a statistical power to distinguish racial and ethnic differences. This study has several limitations. The cross‐sectional design in determining characteristics and clinical covariates was not updated over the study period; thus, we did not account for participant changes in health behavior (e.g., smoking cessation, dietary and exercise changes) and overall health that could have influenced mortality. We adjusted for multiple CVD risk factors that have been shown to affect survival; however, we did not adjust for all possible covariates, and confounding remains a possibility. There were also several covariates included that could have changed with time (e.g., improvement of systolic blood pressure or cholesterol values with lifestyle modification). Additionally, the participants’ history was self‐reported, subject to recall bias, and the classification of race does not account for those of bi‐racial background. Lastly, CVD mortality was determined by *International Classification of Diseases* codes and matching participant identifiers (social security numbers, sex, date of birth), which may have been inadvertently misclassified.

In conclusion, we identified that among a diverse population of US adults without baseline CVD, Mexican Americans had a lower prevalence of SCMI and an increased risk of CVD mortality when compared to other race/ethnicities. These results implicate that addressing SCMI in Mexican Americans may enhance prevention and address cardiovascular disparities.

## 5 ETHICS

The data utilized in this study from NHANES III have been approved by the NCHS Research Ethics Review Board, and documented consent was obtained from participants.

## AUTHOR CONTRIBUTIONS

All authors had access to the data and participated in the drafting and submission of this manuscript.

## CONFLICT OF INTEREST

The authors have no conflicts of interest to disclose in connection with this article.
